# Substitution of warthog NF-κB motifs into RELA of domestic pigs is not sufficient to confer resilience to African swine fever virus

**DOI:** 10.1038/s41598-020-65808-1

**Published:** 2020-06-02

**Authors:** Stephen McCleary, Rebecca Strong, Ronan R. McCarthy, Jane C. Edwards, Emma L. Howes, Lisa M. Stevens, Pedro J. Sánchez-Cordón, Alejandro Núñez, Samantha Watson, Alan J. Mileham, Simon G. Lillico, Christine Tait-Burkard, Chris Proudfoot, Maeve Ballantyne, C. Bruce A. Whitelaw, Falko Steinbach, Helen R. Crooke

**Affiliations:** 1 Virology Department, Animal and Plant Health Agency, APHA-Weybridge, Woodham Lane, New Haw, Addlestone, KT15 3NB UK; 2 Pathology Department, Animal and Plant Health Agency, APHA-Weybridge, Woodham Lane, New Haw, Addlestone, KT15 3NB UK; 3 Animal Science Unit, Animal and Plant Health Agency, APHA-Weybridge, Woodham Lane, New Haw, Addlestone, KT15 3NB UK; 4 Genus PLC, 1525 River Road, DeForest, Wisconsin, 53532 USA; 5 The Roslin Institute, Royal (Dick) School of Veterinary Studies, University of Edinburgh, Easter Bush, Midlothian, EH25 9RG UK; 6 Present Address: Division of Biosciences, Department of Life Sciences, College of Health and Life Sciences, Heinz Wolff Building, Kingston Lane, Brunel University London, Uxbridge, United Kingdom; 7 Present Address: The Pirbright Institute, Pirbright, United Kingdom

**Keywords:** Pathogens, Virology, Agricultural genetics

## Abstract

African swine fever virus (ASFV) causes a lethal, haemorrhagic disease in domestic swine that threatens pig production across the globe. Unlike domestic pigs, warthogs, which are wildlife hosts of the virus, do not succumb to the lethal effects of infection. There are three amino acid differences between the sequence of the warthog and domestic pig RELA protein; a subunit of the NF-κB transcription factor that plays a key role in regulating the immune response to infections. Domestic pigs with all 3 or 2 of the amino acids from the warthog RELA orthologue have been generated by gene editing. To assess if these variations confer resilience to ASF we established an intranasal challenge model with a moderately virulent ASFV. No difference in clinical, virological or pathological parameters were observed in domestic pigs with the 2 amino acid substitution. Domestic pigs with all 3 amino acids found in warthog RELA were not resilient to ASF but a delay in onset of clinical signs and less viral DNA in blood samples and nasal secretions was observed in some animals. Inclusion of these and additional warthog genetic traits into domestic pigs may be one way to assist in combating the devastating impact of ASFV.

## Introduction

African swine fever virus (ASFV) is a large DNA virus and sole member of the family *Asfarviridae* that causes a mostly lethal haemorrhagic disease, African swine fever (ASF), in domestic pigs and Eurasian wild boar. ASFV can genetically be separated into 24 genotypes that cause the same disease, but immunological cross-protection is limited and poorly understood^1^. The introduction of ASF into a country results in trade restrictions and pig losses, thus the disease has a high socioeconomic consequence for both commercial and backyard farmers^2^. Accordingly, the spread of this disease is a serious concern for the global pig industry. Following the incursion of a genotype II ASFV into the Caucasus in 2007 the virus has spread through Russia, entered the European Union in 2014 and, in 2018, was detected for the first time in China. Since then the Chinese pig population has declined by at least 20% and ASFV has further spread across many countries in South East Asia^3,4^. Combating this global threat is hampered by the lack of a vaccine and is particularly difficult in production systems with poor biosecurity which are more vulnerable to virus introduction and contact with wild suids^1^.

ASFV infects all members of the family *Suidae*, which as well as domestic pigs (*Sus scrofa domesticus*), wild boar (*Sus scrofa spp*) and others also includes bushpigs (*Potamochoerus spp*.) and warthogs (*Phacochoerus spp*.), which are considered natural reservoir hosts^5^. In domestic pigs and wild boar acute and subacute forms dominate, but peracute and chronic forms of ASF can occur and the clinical outcome varies accordingly. This clinical outcome is determined by the virulence of the strain, dose and host factors^6^. In acute ASF mortality is >98% with clinical signs developing after a 3–5 day incubation period with death from 7–13 days post infection^7^. The subacute form of ASF has similar but less intense signs with lower mortality rates and death within 15–45 days. With lower virulent strains animals can survive the infection with virus persisting in lymphoid tissues, and survivors can infect naive contact animals^8^, but the extent to which long-term healthy survivors contribute to disease epidemiology is debated^9^.

ASF in warthogs and bushpigs in contrast is considered to be a low pathogenic and persistent infection^10^. Few experimental studies in warthog have been reported but none of 11 warthogs inoculated with ASFV had clinical signs or macroscopic lesions and the animals survived for at least 33 days, by which time viraemia had cleared. In contrast, a proportion of domestic pigs fed tissues from these infected warthogs died within 9 days^11^. This suggests that variations in host factors between these genetically similar species influences the outcomes of infection. Little is known about these host factors. However, 15 nucleotide differences, resulting in 3 amino acid changes, have been identified between warthogs and domestic pigs within the *RELA* gene, which encodes a major component of the NF-κB transcription factor^12^. The NF-κB family of transcription factors consist of distinct combinations of proteins that have a critical role in activating immune cells. As such they regulate the responses to infection including the development of T and B cells and orchestrate a diverse range of proinflammatory cytokines and anti-apoptotic proteins^13,14^. During infection, ASFV targets the host’s NF-κB transcription factor. The viral protein A238L shares homology with porcine IκBα and can substitute for this porcine protein, binding to the RELA (p65) subunit of NF-κB and reducing its ability to be activated^15,16^. Accordingly, the differences in the warthog version of this central regulator of innate and adaptive immune responses may represent a host adaptation that contributes to the lack of haemorrhagic fever in warthogs that is seen in domestic pigs. This hypothesis was supported by *in vitro* comparisons of the warthog and domestic pig RELA variants which indicated that although the two variants are expressed at equivalent levels a lower transcriptional activity for the warthog RELA variant was demonstrated using reporter assays^12^. This raised the possibility that introducing the three variant amino acids of the warthog RELA into domestic pigs may confer some resilience to ASFV infection.

As the otherwise closely related warthogs and domestic pigs do not interbreed, gene editing has the potential to confer such genetic variation across species, allowing the targeted introduction of genes or genetic changes that would otherwise be difficult to achieve through conventional methods. Such genetic livestock improvements are a possible way to enhance disease resistance, as recently demonstrated for other important pig diseases, PRRS and TGE^17^. Enhanced disease resistance or resilience increases not only productivity and sustainability, needed to meet the food demands of an increasing global human population, but particularly in the case of ASF would also improve animal welfare.

Some of us previously reported on the generation of pigs, using Zinc-finger nuclease in embryo gene editing, with either 2 or 3 amino acid substitutions that convert the domestic pig RELA alleles to those amino acids present in warthogs^18^. We therefore aimed to test if these changes alter the outcome of ASFV infection and confer disease resilience.

Intramuscular inoculation of ASFV, which results in a robust and consistent infection model, has been widely used for *in vivo* investigations. However, ASFV transmission in domestic pigs is primarily by contact with virus via direct animal contact or ingestion of infected material^19^. Accordingly, the intramuscular inoculation route bypasses many of the host′s immune defences that would occur on interaction with mucosal surfaces during natural infections. Although potentially less consistent, intranasal inoculation models natural infections^20,21^.

The majority of ASFV strains isolated that are derived from the genotype II virus introduced into Georgia are highly virulent viruses with inoculated animals rapidly succumbing to disease^22^. Whilst it is the spread of these genotype II viruses that is currently causing greatest concern to global pig populations, their high virulence limits the opportunity for assessing subtle effects. For the current studies we accordingly used a genotype X strain (Ken05/Tk1) isolated in Kenya and described as moderately virulent^23^. We therewith established an intranasal inoculation model to subsequently assess the effects of gene editing on ASFV infection in domestic pigs by converting the three amino acids of the domestic pig RELA protein to those encoded by the warthog orthologue.

## Results

### Characterisation of the clinical, virological and pathological parameters induced by intranasal inoculation with different doses of the moderately virulent Ken05/Tk1

As viral dose can impact on the proportion of animals becoming infected and the course of disease^20^ the optimum intranasal dose for the Ken05/Tk1 strain was investigated. Previous studies, using intranasal inoculation of highly or moderately virulent ASFV^20,21^, indicate that a dose of 10^2^ HAD_50_ may not infect a high proportion of animals whereas infection with 10^6^ HAD_50_ resulted in short survival times. To identify a dose infecting a high percentage of animals, but with milder severity and slower disease progression, low, medium and high intranasal inoculation doses were assessed. The actual doses inoculated were confirmed by back titration as 4.4 × 10^4^ HAD_50_/pig (high dose) and ten-fold dilutions thereof.

**Figure 1:**
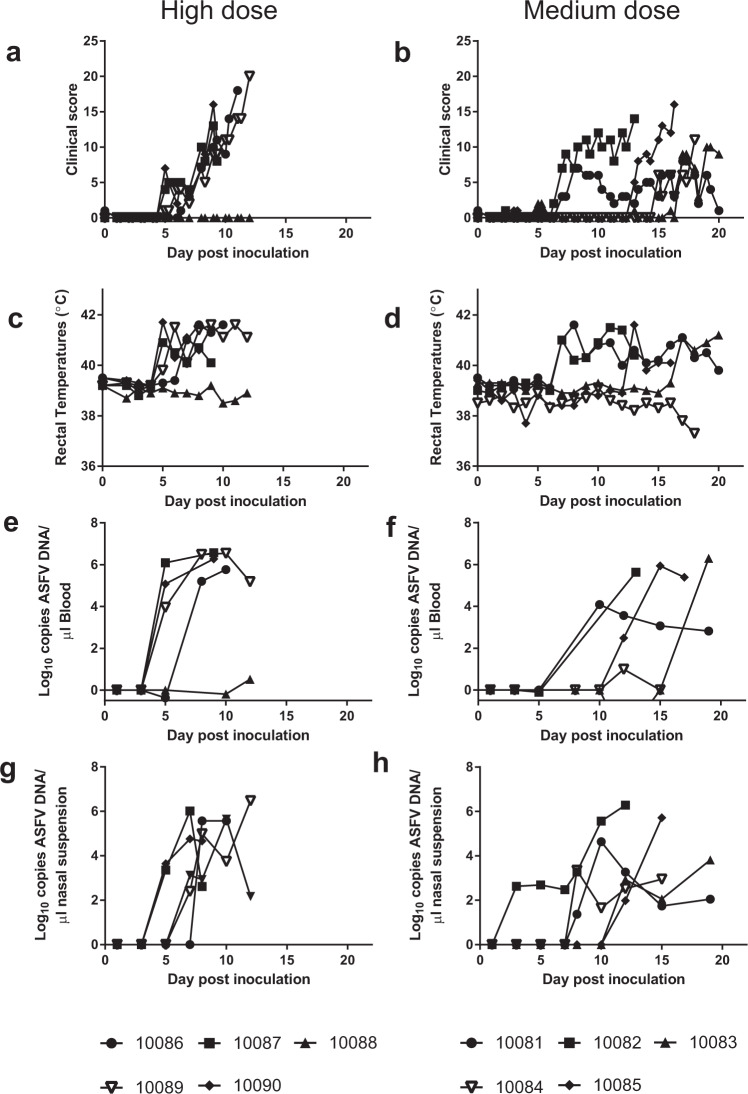
Clinical and virological parameters in pigs inoculated intranasally with medium and high doses of ASFV Ken05/Tk1. Animals inoculated with different doses of virus were observed and clinical scores recorded twice daily (**a,b**). Rectal temperatures were taken once daily (**c,d**). Viral DNA concentrations in blood (**e,f**) and nasal swab samples (**g,h**), taken at 2,3 day intervals, were determined by qPCR.

Clinical signs of increased temperature, lethargy and reduced appetite were first observed in two animals (10087 and 10090) that received the highest dose from 5 days post inoculation (dpi). Signs were observed in two further animals in this group from dpi 6/7. Clinical scores subsequently increased in these animals, which were euthanized between 9 and 12 dpi. No clinical signs were observed in the remaining animal in this group, 10088, which was also euthanized on day 12 (Fig. 1a,c).

Increased temperature, lethargy, reduced appetite and erythema were initially observed in two animals (10081 and 10082) in the medium dose group from 7 dpi, thus slightly later than the high dose group (Fig. 1b,d). One of these animals, 10081, had only mild, fluctuating, clinical signs until the end of the experiment on day 20. This observation is consistent with a subacute, rather than acute, form of ASF. Pig 10082 had clinical signs that indicated a slightly slower progression of disease than animals in the high dose, with fluctuating temperatures but nevertheless had to be euthanized on day 13 post infection. Clinical signs were initially observed in the remaining 3 animals (10083, 10084 and 10085) inoculated with the medium dose at 13, 15 and 17 dpi. Disease then developed rapidly in these animals which were euthanized 3 or 4 days after disease onset. Increases in rectal temperature were consistent with other clinical signs, apart from animal 10084 (Fig. 1d) which showed clinical alterations, including ataxia, but no increased temperature. Weight gain was also consistent with the clinical score and temperature observations with weight gain stopping or decreasing from the onset of clinical signs (Supplementary Table S1)

Animals inoculated with the lowest dose remained healthy throughout the experiment and continued gaining weight. Viral DNA was not detected in the blood or nasal swabs (data not shown) of any animals of this group except for one, possibly erroneous, positive sample. Necropsies only revealed non-specific lesions, which were not suggestive or characteristic of ASF, and thus we conclude the inoculated dose was too low to infect these animals intranasally.

Four of the five animals receiving the highest dose were positive for ASFV DNA in blood at 5 dpi. Of these, pig 10086 had low viral DNA levels (less than 1 copy per μl) in blood at this time (Fig. 1e). Viral DNA in the blood of these animals rapidly reached high levels, coinciding with the rapid onset of clinical signs, indicating these animals were directly infected by the intranasal inoculation. The remaining animal in this group, 10088, was only positive from day 10, after high levels of viral DNA were detected in nasal secretions of pen mates (Fig. 1g), indicating this animal was most likely infected by contact rather than due to a longer incubation period. Low levels of viral DNA were detected in nasal swabs of this animal prior to detection of virus in blood samples. Virus derived from secretions from infected pen-mates is likely to contribute to this low level of viral DNA detected in nasal swabs taken prior to the onset of viraemia.

ASFV DNA was detected first in the blood of animal 10081 in the medium dose group (Fig. 1f). Whilst 10082 had high viral loads in blood and nasal secretions (Fig. 1h) until euthanasia, viral DNA levels in samples from 10081, which had lower clinical signs, declined from 10 dpi. Viral DNA was detected in blood samples of the remaining animals of this group one or 2 weeks later except for pig 10084, which did not have a raised temperature and this animal’s blood samples remained PCR negative apart from one low positive sample on day 12.

**Figure 2:**
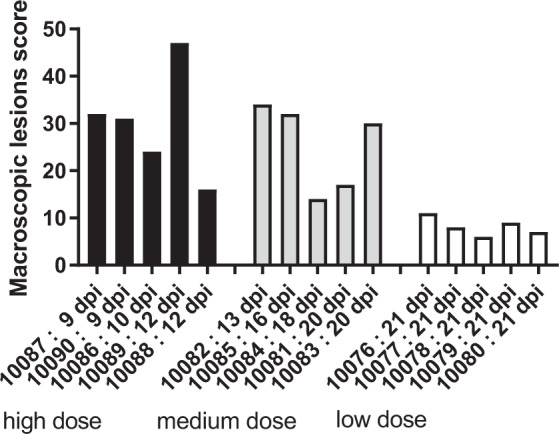
Macroscopic pathological lesions induced by different doses of Ken05/Tk1. Macroscopic lesions observed upon necroscopy of animals inoculated with high, medium and low doses were scored according to severity/number of lesions. The day post inoculation that animals were euthanized is indicated.

Upon post mortem examination all pigs in the high dose group, with the exception of pig 10088, had high macroscopic scores and all had lesions characteristic of ASF, such as cyanotic areas on the skin, ascites, congested lungs with interstitial and alveolar oedema, lymphadenitis with petechial haemorrhages in lymph nodes, and petechial haemorrhages in kidney (Fig. 2).

Pigs inoculated with the medium dose were euthanized between 13 and 20 dpi. Pig 10082 was euthanized at 13 dpi; animals 10085 and 10083, which were probably infected by contact, were euthanized at 16 and 20 dpi respectively. Animals had macroscopic lesions characteristic of ASF similar to those observed in the high dose group. Pig 10081 displayed only mild clinical signs and both 10081 and 10084 had fewer and less severe macroscopic lesions (Fig. 2). Haemorrhagic lymphadenitis for example, suggestive of ASF virus infection, was not observed in pig 10081 and was only observed in one lymph node in pig 10084.

Intranasal inoculation with a dose of 4.4 × 10^4^ HAD_50_ accordingly resulted in direct infection of 80% of animals which developed an acute form of ASF, with consistent clinical, virological and pathological parameters and animals surviving for between 9 and 12 dpi before reaching the predetermined clinical end point. By contrast, only 40% of animals were directly infected by a 10 × lower dose, with a more variable disease outcome. From these data the PID_50_ (dose required to infect 50% of pigs) for intranasal inoculation of the Ken05/Tk1 strain was calculated as ~10^3.9^ HAD_50_/pig.

### Comparison of clinical, virological and pathological parameters induced in pigs with or without warthog motifs of RELA

The first part of the study demonstrated that the higher dose (4.4 × 10^4^ HAD_50_/pig) represented the best experimental model, providing a relatively reliable infection, with animals surviving for 9 to 12 days until euthanasia was required. To assess if the alteration of amino acids in RELA of domestic pigs conferred resilience to ASFV infection, animals with either 2 or 3 amino acid edits (2aa or 3aa), and matched wild type controls were inoculated intranasally with an equivalent dilution of the same ASFV Ken05/Tk1 stock used for the high dose group in the initial experiment. Back titration of the inoculum this time indicated a dose of 1.4 × 10^4^ HAD_50_ per pig was achieved.

**Figure 3:**
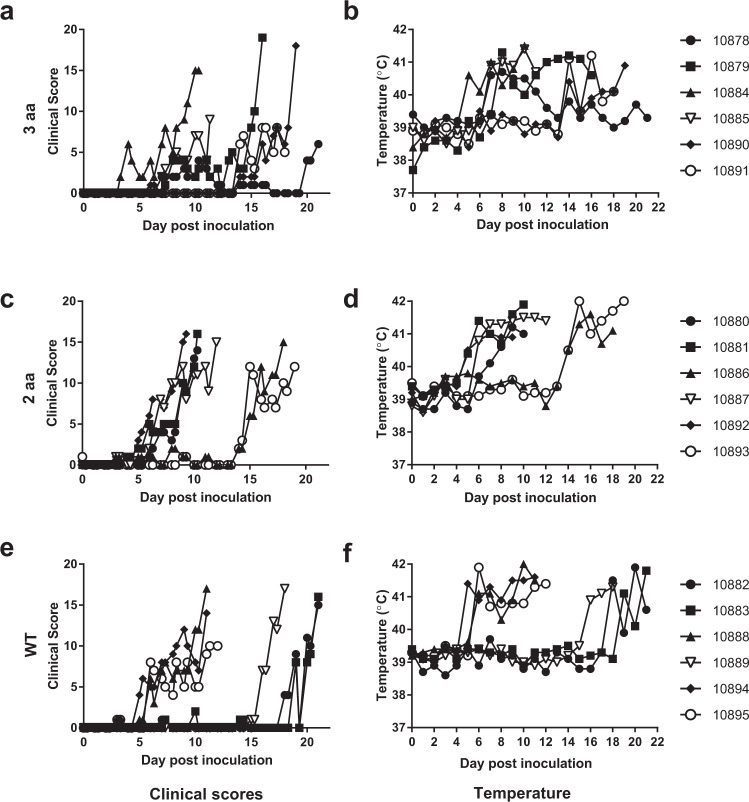
Clinical scores and temperature responses of animals with alterations within RELA upon infection with ASFV. Clinical observations and fever response to intranasal inoculation with Ken05/Tk1 in animals with alterations in 3 amino acid (**a,b**) or 2 amino acid (**c,d**) of the RELA protein and matched wild type control animals (**e,f)**.

**Figure 4:**
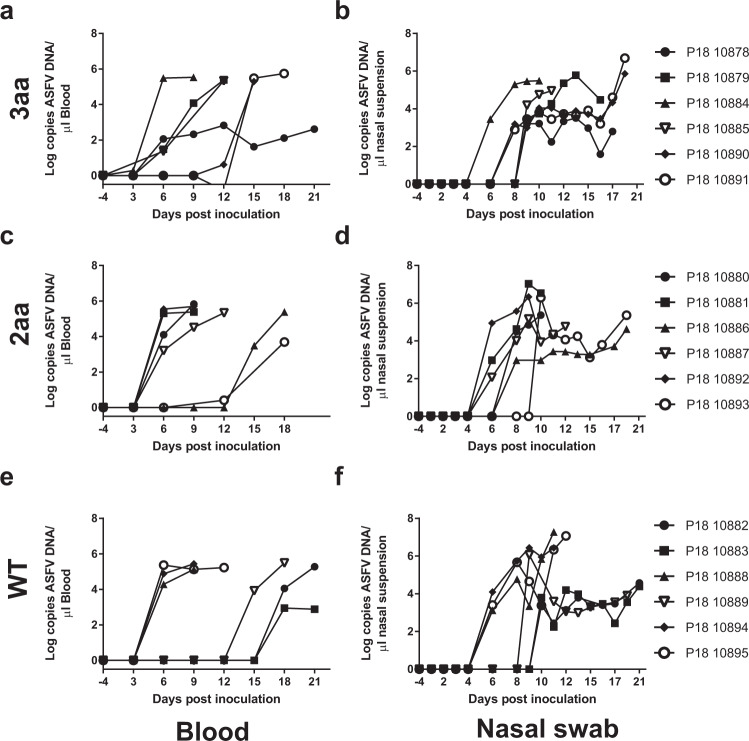
Impact of gene alterations in RELA on kinetics and levels of viraemia and shedding of virus in nasal secretions. Concentrations of ASFV DNA in blood and nasal swab suspensions taken from animals with three (**a,b**) or two (**c,d**) amino acid alterations in RELA and wild type animals (**e,f**) were determined by qPCR.

Clinical alterations were initially observed in pig 10884, in the 3aa group, which had severe diarrhoea, reduced appetite and lethargy at 4 dpi and was treated with rehydration therapy. These early signs may have been due to factors other than the ASFV infection as this animal did not have an elevated temperature at this time (Fig. 3a,b). Signs were next observed between 5 to 7 dpi, in 4 of the 6 animals in the 2aa group (Fig. 3c,d) and 3 of 6 in the WT group (Fig. 3e,f). Apart from 10884 there was a slight delay in onset of clinical signs in animals in the 3aa group, with a further 3 animals (10878, 10879 and 10885) having signs from 7 dpi (Fig. 3a,b). Clinical signs then increased rapidly in the 4 animals from the 2aa group and the 3 animals in the WT group. These signs were associated with an increase in temperature above 41 °C (Fig. 3d,f) and either a decrease in weight or a cessation of weight gain (Supplementary Table S2) and high viral loads in blood (Fig. 4c,e). These 7 animals were euthanized upon reaching the predefined clinical end point between 9 and 12 dpi. Of the animals with early clinical signs within the 3aa group animal 10884 rapidly developed high clinical scores with a high temperature. This animal stopped gaining weight, had high viral loads in blood and was euthanized at 10 dpi. Pig 10885 from the 3aa group also had increased temperature at 7 dpi, a rapid increase in clinical signs and had high levels of DNA in blood on 11 dpi when this animal was euthanized prior to reaching the clinical end point due to uncontrolled epistaxis (nose bleed). These observations are consistent with all these 9 animals having developed an acute form of ASFV from the inoculum.

A slightly different course of disease was observed in animals 10878 and 10879 in the 3aa group. These animals had clinical signs (increased temperature and slight conjunctivitis in 10879) that resulted in only low clinical scores from 7 dpi. The initial increase in temperature then decreased in both animals at 9 dpi and clinical scores remained low, with occasional clinical scoring due to slightly reduced liveliness. After a period with a lower temperature pig 10879 again had an increased temperature and subsequently had reduced liveliness, stiffness when standing and reduced appetite from 15 dpi and was euthanized at dpi 16. In contrast, animal 10878 maintained a lower, fluctuating, temperature from 13 dpi, but had only few clinical signs and continued to gain weight (although at a reduced rate compared to unaffected animals). This animal was euthanized at the end of the experiment, when incidentally some increase in clinical scores were observed just before euthanasia. In contrast to other animals, pig 10878 consistently had a low level of viral DNA in blood (Fig. 4a) and DNA levels in nasal secretions remained low and at a level that may be due to viral DNA derived from pen-mates rather than due to secretion by pig 10878 (Fig. 4b,d,f).

To assess if the amino acid changes within RELA impacts the host′s pro-inflammatory response to ASFV, serum cytokine levels of IL8, TNF-α, IL1-β and IL-6 were assessed. Cytokine responses were variable in the different animals, and relatively low, with an increase that corresponded to progression of clinical disease only being observed in some animals from each group (Supplementary Figs. S2 and S3). There was no indication that either the 2 or 3 amino acid alterations within RELA impacted the host′s response to ASFV infection for any of these pro-inflammatory cytokines.

Interestingly, apart from animal 10884 which had clinical signs characteristic of an enteric bacterial infection at day 4, the viral loads in blood of 3 of the 4 animals in the 3aa group (10878, 10879 and 10885) that became infected early were lower than in early infected animals in the WT or 2aa group at 6 days post infection (Fig. 4a,c,e). Comparison of the DNA levels of all early infected animals in each group at this time point does not reveal a statistically significant difference. However, the difference in viral DNA levels between the 3aa group and the WT or 2aa group is significant (P = 0.004) at this time point if 10884, which may have been compromised due to a concurrent infection, is omitted. Similarly, with the exception of 10884, a lower level of viral DNA was detected in nasal secretions from animals in the 3aa group at day 6 and 8 pi than in the 2aa or WT animals (P < 0.05 if 10084 omitted) (Fig. 4b,d,f).

The remaining 7 animals (2 in both gene edited groups and 3 in the wild type group) developed clinical signs, had increased temperature and viral DNA in blood on or after 14 dpi, after high levels of viral DNA was detected in nasal swabs of animals within the same room, indicating that these animals became infected through contact infection. All of these contact infected animals, including the two in the 3aa group, had a rapid increase in clinical score, high temperatures, reduced weight gain and high viral loads in blood, consistent with an acute course of disease.

As was also observed in the initial study total leukocytes counts did not indicate that leukopenia was correlated with the development of clinical disease. However, a decrease in lymphocytes was observed in animals where other parameters indicated an acute course of disease. Lymphocyte numbers decreasing from 3 dpi onwards and approached or dropped below 50000 cells/ml in animals that were euthanized between 9 and 12 dpi. Lymphocyte numbers decreased in pig 10878, but less rapidly than the other early affected animals, and cell counts in this animal began to recover after 13 dpi when its temperature had also reduced (supplementary Fig. S1).

**Figure 5:**
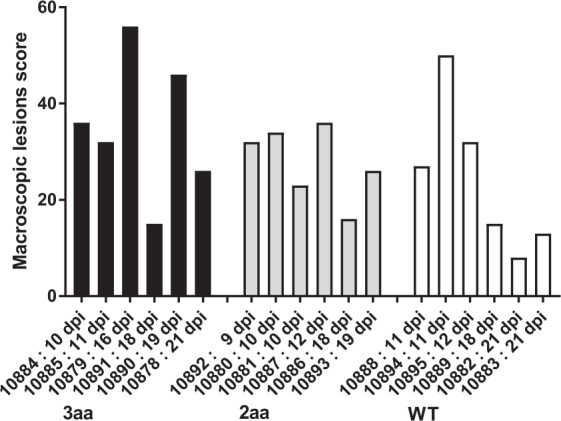
Impact of gene alterations in RELA on pathological lesions induced by ASFV. Macroscopic lesions observed upon necroscopy in animals with 2 or 3 amino acid alterations in RELA or wild type controls scored according to severity/number of lesions. The day post inoculation that animals were euthanized is indicated.

Upon post mortem examination all pigs had macroscopic lesions characteristic of ASF such as described in the pilot experiment (Fig. 5). Macroscopic lesion scores were highest in two pigs within the 3 aa group that were euthanized from dpi 16 onwards (10879 and 10890) and in one pig in the WT group (10894) euthanized at 11 dpi. Further to lesions characteristic of ASF, pig 10884 (3aa) had a diffuse severe diphtheroid enteritis that affected jejunum and ileum, lesion characteristic of a bacterial co-infection, supporting the notion that this pig had a pre-existing condition.

Pig 10878 (3aa group) that had a clinical score of 6 at euthanasia had more severe macroscopic lesions than those observed in some of the animals infected by contact infection at a later stage, such as 10091, 10086, 10089, 10082 and 10083, which had higher clinical scores and viremia levels at time of euthanasia but which had been infected for a shorter time.

Statistical analysis of macroscopic scores did not reveal significant differences among experimental groups.

## Discussion

The devastating spread of genotype II ASFV in pig producing regions across the world, and the continuing presence of other genotypes in Africa and Sardinia, highlight the requirement for increased research efforts to find disease control options. Such research requires a range of appropriate and well characterised *in vivo* challenge models for the assessment of methods to combat infection. Whilst many recent studies understandably use experimental challenge models based upon the genotype II viruses currently impacting pig producers in Europe and Asia, these viruses are highly virulent^24^. This limits the assessment of interventions that may have some promise, but for which the benefits may not be apparent under such challenge conditions. In addition the continued presence of other ASFV genotypes in Africa necessitates challenge models using strains of diverse genotypes for the assessment of novel interventions that could permit the development of the African pig industry to its full potential^25^. Experimental models using intramuscular inoculations have the advantage of consistency, thereby reducing experimental variation and hence group sizes. However, the intramuscular route bypasses the initial mucosal immune surfaces encountered by the pathogen, results in differences in viraemia and shedding profiles compared to contact infection and is therefore a suboptimal method for assessing interventions that may act at early stages of a natural infection^20,26^.

We therefore sought to characterise the clinical, virological and pathological parameters induced by a less virulent, genotype X virus when inoculated via the intranasal route. The Ken 05/Tk1 isolate is considered moderately virulent as a percentage of animals were able to survive after intramuscular inoculation, with a dose of 10 HAD_50_, for at least 70 days^23^. As expected a higher dose was required for infection via the intranasal route compared to intramuscular route. The calculated PID_50_ of ~10 ^3.9^ HAD_50_ was consistent with other studies using intranasal, intraoropharyngeal or intranasalpharyngeal routes of inoculation, with virulent or moderately virulent strains^20,27^.

In the group infected with the medium dose (4.4 × 10^3^ HAD_50_) only 2 of 5 animals were infected directly and one of these (10081) had a subacute form and may have gone on to survive the infection if a longer timeframe were allowed. However, all animals infected in the high dose group developed an acute form but clinical signs were such that animals only required euthanasia after 9–12 days. Using this strain intranasally at a dose of 4.4 × 10^4^ HAD_50_ therefore represents a less virulent challenge model than intramuscular inoculation of highly virulent strains and results in a consistent disease course in the majority of animals. Accordingly and noteworthy, an increased chance of subacute disease and a lower chance of infection were observed with the lower 4.4 × 10^3^ HAD_50_ dose.

The dose required to cause infection via natural routes is an important parameter for risk models of transmission^28^ and this data provides additional information on the dose required to infect animals by transmission routes that may expose virus to the nasopharynx. Interestingly, the determined PID_50_ dose was less than the median infective dose observed when feeding ASFV contaminated feedstuffs but is higher than the amount of virus sufficient to initiate infection via liquid intake^29^. This highlights the requirement for careful consideration of all potential transmission routes and potential dose variations during risk assessment.

Technology to generate gene edited livestock including pigs is developing rapidly and represents a method to improve disease resistance. Many gene editing approaches target host cell receptors as a method to block virus entry and prevent infection. For example, pigs with alterations in the CD163 gene are fully resistant to infection with PRRSV^17^. Unfortunately, what works for one disease may not work against another, particularly if viruses use different cell receptors and CD163 edited pigs were not resistant to ASFV infection^30^.

Targeting the host′s immune defences, such as the NF-κB master regulator of inflammatory responses represents an alternate strategy for the generation of livestock better able to cope with a range of infectious diseases.

No differences were detected in any parameter measured between the wild type animals and those containing the 2aa substitutions at T448A and S485P, indicating that these two differences in the warthog RELA protein, which are both located in transactivation domain 2 and hence could be involved in interactions within the transcriptional complex, have no impact on the outcome of ASFV infection.

The additional S531P substitution in the animals of the 3aa group is located in transactivation domain 1 of RELA and is predicted to undergo phosphorylation^12^. Transient transfection of NF-κB -luciferase reporter cells with RELA variants containing the individual amino acid substitutions indicated that the majority of the reduced transcription activation activity observed for the warthog RELA is due to the change at this position^12^. Disappointingly all of the animals with the three amino acid substitutions developed clinical signs of ASF, after either direct infection by the inoculum or by later contact infection, and 5 of the 6 had to be euthanized prior to the end of the experiment. These 3 changes are therefore insufficient to confer the level of resilience to infection observed in warthogs upon domestic pigs. However, 3 of 4 animals with the 3 amino acid substitution that were infected directly by the inoculation had slightly delayed clinical signs and lower levels of viral DNA in blood and nasal secretions, and one of these animals developed subacute rather than acute disease.

Although we cannot exclude the conclusion that the observations are due to the inherent variability of the infection model, it is tempting to speculate that the substitutions, particularly the serine to proline modification at position 531, may have given the 3aa animals a slight advantage of delayed virus replication and increased the chance of a subacute rather than acute infection. We did not detect any clear impact of the alterations in RELA on the induction of pro-inflammatory cytokines in response to ASFV infection which could imply that modulation of the pro-inflammatory response is not involved in this potential delay in virus replication. However, the level of induction of these cytokines was relatively low even in the wild type animals which may be a reflection of the moderate virulence of the Ken05/Tk1 strain. Further investigations would be required to identify the impact of the RELA changes on the myriad of NF-κB mediated immune responses.

There was no significant reduction in lesions post mortem indicating that any advantage conferred that may reduce early clinical signs or viraemia was not correlated to organ changes at the time of euthanasia. Also no similar delay in signs or reduction in viral loads was observed in the two contact infected animals, indicating these changes in RELA were unable to abate viral replication after contact infection and that any effect is only pertinent at low dose infections at the start of an infection chain. The initial introduction of virus onto a premise can occur via fomites or ingestion of contaminated material which may only contain low doses of virus. Weak or runted animals are known to be susceptible to even very low oronasal doses and have been suggested may act as amplifiers that allow infection to establish on a premise^31^. A pig breed which can reduce or delay early virus replication might be better placed to resist a low dose exposure and so reduce the chances of disease establishing.

The alterations in warthog RELA have the potential to affect the host′s response to ASFV in a variety of ways. The reduced basal transcriptional activity of warthog RELA demonstrated by Palgrave *et al*. (2011) could modify the pattern of expression of a vast number of genes in different tissues that could lead to a potential advantage for the host and might explain the reduced clinical signs. A238L is a potent anti-inflammatory viral protein^32^ but mutant ASFV, in which the *A238L* gene is deleted, demonstrated that it is not essential for viral growth in macrophages, nor were differences observed upon infection with these A238L deleted viruses *in vivo*^33,34^ implying the absence of a substantial role of A238L in ASFV virulence.

For a tool to combat ASFV the ideal breed improvement would be pigs that are resistant to infection (the ability to prevent the establishment or development of infection). In this case we sought to examine if the gene edits would result in an improved resilience to infection. Animals that are resilient remain productive in the presence of infection and would be beneficial to allow development of the African pig industry, which has long been hampered by the wildlife reservoirs of ASFV, and also for those countries facing the current disastrous impacts of the disease that will be challenging to control, especially if the disease becomes endemic.

A drawback of disease resilient animals is that they may harbour virus, but present with few clinical signs. This would make early detection and rapid control of disease outbreaks more difficult and long term shedding of virus by resilient animals that go undetected could contribute to disease transmission. The current study did not result in animals with the level of resilience observed in warthogs. At present warthog genome sequence data in the public databases are limited to short read sequences representing about 30x genome coverage^35^. However, a highly contiguous genome assembly for the common warthog (*Phacochoerus africanus*) has been reported recently^36^. This warthog reference genome sequence, which is being polished prior to release into the public sequence databases (A.L. Archibald, pers comm), will facilitate the identification of additional warthog alleles that could be beneficial for ASFV resilience. If successfully developed, resilient animals would have the potential to benefit animal welfare but could also contribute to disease spread and therefore would be unlikely to be considered desirable in many sectors of the global pork production industry. However, such animals could represent a lifeline to those farmers in resource-poor settings facing an onslaught of ASFV, for example from endemically infected wildlife populations, where the value of an individual animal is high. The responsible use of such resilient animals would need to be accompanied by robust efforts and legislation by individual nations to improve and maintain good biosecurity as well as implementation of enhanced disease surveillance, to prevent unintended increase in disease transmission.

Whilst the tested amino acid substitutions on their own did not convey sufficient protection the potential for an impact on the virus raises the possibility that pigs modified to include these, along with other as yet uncharacterised differences in the resilient warthog, warrant further investigation and may be one way to contribute to the multitude of efforts required to reduce the substantial financial losses and animal welfare impacts occurring due to ASF.

## Methods

### Virus

ASFV Ken05/Tk1, a genotype X virus originally isolated from a tick was provided by the EU Reference Laboratory for ASF (CISA-INIA, Valdeolmos, Madrid, Spain). A stock for inoculations was grown in primary porcine peripheral blood monocytes and viral titres were determined as the amount of virus causing haemadsorption in 50% of inoculated cultures (HAD_50_/ml). Freedom from other porcine pathogens was confirmed by analysis of viral nucleic acid extracted from stocks on a pan viral microarray^37^.

### Animals and experimental design

Production of founder pigs with edits to the RELA gene conferring 2aa or 3aa substitutions was reported previously^18^. In addition to the intended gene edits, two of the three founder boars were known to also contain random integration of the plasmid used as HDR template. Founders were crossed with wild type females and offspring genotyped. Individuals with the desired gene edits and absence of the plasmid were crossed to produce homozygous (2aa or 3aa) or wild type littermates for the RELA study. To ensure both studies were completed in the same genetic background wildtype siblings generated from these crosses were used for the initial model development study.

Animal were housed on straw within isolation rooms in animal containment facilities at the Animal and Plant Health Agency (APHA, Weybridge, UK) licenced for ASFV. Studies were reviewed by the APHA animal welfare and ethical review board and conducted in accordance with the UK Animals (Scientific Procedures) Act 1986 under project licence PF971B5E3

For characterisation of the intranasal inoculation model, 15 wild type animals were grouped into 3 groups of 5. Animals were assigned so that the groups had similar mean weight and distribution of male and female animals per group. Animals were inoculated at 12 weeks of age, after a period of acclimatisation, using intranasal mucosal atomisation devices (MAD Intranasal, Teleflex), with 2 ml (1 ml per nostril) of Ken05/Tk1 virus. All dilutions of the inocula for the different doses were titrated on peripheral blood monocyte/macrophages on the day of inoculation to confirm the actual HAD_50_ dose administered.

To assess the impact of alterations within RELA three groups of six, 10-week-old animals were inoculated intranasally with a target dose of 4.4 × 10^4^ HAD_50_ Ken 05/Tk1. The first group had 3 altered amino acids (3aa) within the RELA protein, the second group had 2 amino acids altered (2aa) and the third group (WT) had the domestic pig wild type sequence. To minimise the potential for room effects and allow for blinding of study groups, animals were housed in 3 rooms each containing 6 pigs, with 2 pigs per group in each room.

### Sampling and clinical observations

Animals were assessed for clinical signs twice daily, using a slightly modified version of the scheme describe previously^38^ that assesses 10 parameters relevant to an indication of swine fever (rectal temperature, liveliness, body shape and tension, breathing, walking, skin, eye/conjunctiva, appetite and defecation) and scored as 0 (normal), to 3 (severely altered). Animals were euthanized by overdose of barbiturate upon reaching a pre-defined clinical score humane end point, at the end of the experiment or to prevent housing of single animals. Rectal temperatures and weights were taken once daily. Blood samples were taken from the anterior vena cava prior to inoculation, then at 2 or 3 day intervals post inoculation (dpi) into EDTA vacutainers (BD biosciences). Nasal swabs were taken at 1–3 day intervals and re-suspended in 1 ml of PBS.

### Quantification of viral DNA levels in blood and nasal swabs and haematological and pathological observations

Viral nucleic acid was extracted from blood and nasal swab suspensions using Viral RNA mini kit (Qiagen) and viral DNA levels (genome copies) determined by real time PCR, as described, with the exception that primers and probes to detect CSFV were excluded^39^. The number of copies of viral genome were quantified by comparison to standards of known concentrations of a plasmid, pVP72, encoding the target region of the ASFV genome. Leukocytes present within 25 μl of EDTA blood were detected by staining with 2.5 μl of a porcine pan-leukocyte anti-CD45-FITC antibody (AbD Serotec). Samples were then mixed with 475 μl FACS lysing solution (BD Biosciences), prior to dilution with 2 ml of PBS and assessment of total leukocyte and lymphocyte counts in 200 μl of the cell suspension using a GUAVA flow cytometer. The level of cytokines in serum samples were determined by porcine TNFα DuoSet and porcine IL-6, IL-8 and IL-1β Quantikine ELISAs (R & D systems). During necropsies, macroscopic lesions were evaluated following method based on previous standardized protocols^7^.

The intranasal PID_50_ (dose required to infect 50% of pigs via intranasal route) was calculated using the Spearman & Kärber formula.

Statistical difference between viral loads was determined using Graphpad Prism by one way Anova with tukey’s multiple comparison test.

## Supplementary information

Supplementary information.
