# Supplementary material for: Substitution of warthog NF-κB motifs into RELA of domestic pigs is not sufficient to confer resilience to African swine fever virus
Source: Sci Rep. 2020 Jun 2;10:8951. doi: 10.1038/s41598-020-65808-1 (PMC7265332; doi:10.1038/s41598-020-65808-1)
Supplement: Supplementary file 1 — Supplementary information. [file 41598_2020_65808_MOESM1_ESM.docx]

**Substitution of warthog NF-κB motifs into RELA of domestic pigs is not sufficient to confer resilience to African swine fever virus**

Stephen McCleary^1^, Rebecca M. Strong^1^_,_ Ronan R. McCarthy^1‡^, Jane C. Edwards^1‡‡^, Emma L. Howes^1^, Lisa M. Stevens^1^_,_ Pedro J. Sanchez-Cordon^2^, Alejandro Nunez^2^, Sam Watson^3^, Alan J. Mileham^4^, Simon G. Lillico^5^, Christine Tait-Burkard^5^, Chris Proudfoot^5^, C. Bruce A. Whitelaw^5^, Falko Steinbach^1^  & Helen R. Crooke^1^*

Supplementary Table 1: Mean daily weight gain in animals inoculated with low, medium or high doses of ASFV Ken05/Tk1

| Dose | Animal ID | Mean daily weight gain Kg | | | |
| --- | --- | --- | --- | --- | --- |
|  |  | Dpi 1-5 | Dpi 6-10 | Dpi 11-15 | Dpi 15-20 |
| Low | 10076 | 0.4 | 1 | 0.4 | 0.6 |
|  | 10077 | 0.8 | 1 | 0.4 | 0.6 |
|  | 10078 | 0.8 | 1 | 0.4 | 0.8 |
|  | 10079 | 0.4 | 1 | 0.4 | 0.8 |
|  | 10080 | 0.2 | 1.4 | 0 | 0.4 |
| Medium | 10081 | 1.2 | -0.2 | 0.8 | 0 |
|  | 10082 | 1.4 | -0.8 | -1 |  |
|  | 10083 | 1.6 | 1 | 1.2 | -0.8 |
|  | 10084 | 0.4 | 0.6 | 0.4 | -0.5 |
|  | 10085 | 1.6 | 1.2 | -0.8 | 0 |
| High | 10086 | 0.7 | -0.2 |  |  |
|  | 10087 | 0.3 | -0.7 |  |  |
|  | 10088 | 1 | 1.4 | 0.5 |  |
|  | 10089 | 1.3 | -0.6 | 0 |  |
|  | 10090 | 0.6 | -0.5 |  |  |

Supplementary Table 2: Mean daily weight gain in gene edited or wild type control animals inoculated with ASFV Ken05/Tk1

| Group | Animal ID | Mean daily weight gain Kg | | | |
| --- | --- | --- | --- | --- | --- |
|  |  | Dpi 1-5 | Dpi 6-10 | Dpi 11-15 | Dpi 15-20 |
| 3aa | 10878 | 0.8 | 0.4 | 0.6 | 0.4 |
|  | 10879 | 0.5 | 0.4 | 0.0 | 0.0 |
|  | 10884 | 0.3 | -0.4 |  |  |
|  | 10885 | 0.8 | 0.6 | -1.0 |  |
|  | 10890 | 0.8 | 0.6 | 0.6 | -0.3 |
|  | 10891 | 0.8 | 0.6 | 0.4 | 0.3 |
| 2aa | 10880 | 0.8 | 0.0 |  |  |
|  | 10881 | 0.5 | -0.2 |  |  |
|  | 10886 | 0.8 | 1.0 | 1.0 | -1.3 |
|  | 10887 | 0.5 | -0.2 | -0.5 |  |
|  | 10892 | 1.0 | -0.8 |  |  |
|  | 10893 | 0.8 | 0.8 | 1.2 | -0.3 |
| WT | 10882 | 1.5 | 1.0 | 1.0 | 0.0 |
|  | 10883 | 0.5 | 1.0 | 1.0 | 0.2 |
|  | 10888 | 0.8 | 0.0 |  |  |
|  | 10889 | 1.0 | 1.2 | 1.0 | -1.0 |
|  | 10894 | 1.0 | -0.4 | 0.0 |  |
|  | 10895 | 0.8 | -0.2 | -0.5 |  |
